# Direct Matrix-Assisted Laser Desorption Ionization Time-of-Flight Mass Spectrometry Improves Appropriateness of Antibiotic Treatment of Bacteremia

**DOI:** 10.1371/journal.pone.0032589

**Published:** 2012-03-16

**Authors:** Anne L. M. Vlek, Marc J. M. Bonten, C. H. Edwin Boel

**Affiliations:** Department of Medical Microbiology, University Medical Center Utrecht, Utrecht, The Netherlands; Statens Serum Institute, Denmark

## Abstract

Matrix assisted laser desorption ionization time-of-flight mass spectrometry (MALDI-TOF MS) allows the identification of microorganisms directly from positive blood culture broths. Use of the MALDI-TOF MS for rapid identification of microorganisms from blood culture broths can reduce the turnaround time to identification and may lead to earlier appropriate treatment of bacteremia. During February and April 2010, direct MALDI-TOF MS was routinely performed on all positive blood cultures. During December 2009 and March 2010 no direct MALDI-TOF MS was used. Information on antibiotic therapy was collected from the hospital and intensive care units' information systems from all positive blood cultures during the study period. In total, 253 episodes of bacteremia were included of which 89 during the intervention period and 164 during the control period. Direct performance of MALDI-TOF MS on positive blood culture broths reduced the time till species identification by 28.8-h and was associated with an 11.3% increase in the proportion of patients receiving appropriate antibiotic treatment 24 hours after blood culture positivity (64.0% in the control period versus 75.3% in the intervention period (p0.01)). Routine implementation of this technique increased the proportion of patients on adequate antimicrobial treatment within 24 hours.

## Introduction

Bloodstream infections are associated with high rates of morbidity and mortality and microbiological identification of causative pathogens is crucial for optimal management[1]. However, conventional identification is based on time-consuming procedures, and species identification and susceptibility testing usually requires at least 48 hours after blood cultures become positive.

Developed in the 1980s [2], [3], the MALDI technique is an ionization technique that allows the analysis of biomolecules. Ionized biomolecules are accelerated in an electric field, and different molecules are separated according to their mass to charge ratio. The method is used for detection and characterization of biomolecules with molecular masses between 400 and 350,000 Da. The feasibility of MALDI-TOF MS identification of bacterial colonies from solid media has been assessed on a wide array of clinically relevant bacterial strains as well as yeast isolates. Correct identification at the species level is obtained in 80–95% of bacterial isolates [4], and in 66–87% when applied directly on positive blood culture broths [5]–[8].

Faster identification of microorganisms causing bacteremia could allow appropriate species-specific therapy to be started sooner, thereby improving patient outcome and reducing potential development of resistance and possible side effects [9]. Direct application of MALDI-TOF MS on blood culture broth samples after growth identification of automated culture methods (such as the Bactec 9240 system (Becton-Dickinson, Sparks, USA)) might allow earlier implementation of appropriate antimicrobial treatment, but has not been investigated in real-life settings. The aim of this prospective clinical trial was to assess the impact of performing direct MALDI-TOF MS on positive blood cultures on turnaround time and antimicrobial management in patients with bloodstream infections.

## Methods

### Study design

The study was conducted between December 2009 and April 2010 in the University Medical Center Utrecht. Direct MALDI-TOF MS was routinely performed on all consecutively positive blood cultures during February and April 2010, and standard care procedures were used in December 2009 and March 2010. In January 2010, technicians were trained in performing direct MALDI-TOF MS analysis. Direct MALDI-TOF MS was performed twice daily on weekdays and once daily during weekends. All episodes of positive blood cultures, based on alerts of the Bactec 9240 system, were included. One episode included all positive blood cultures with the same pathogen(s) within one week in one patient. Cultures were defined as contaminated if Coagulase-negative staphylococci or other cutaneous flora was cultured and not considered clinically relevant by the treating physician. Susceptibility testing was performed using the Phoenix system (Becton-Dickinson, Sparks, USA) during the total study period. Results of susceptibility testing required on average 48 hours after blood cultures became positive. In the standard care group, antibiotic treatment could be adjusted based on the information that was available from Gram staining, culturing results and results of susceptibility testing. In the intervention group, antibiotic treatment could be adjusted based on the results of direct MALDI-TOF MS analysis in addition to the information that was available from Gram staining, culturing results and results of susceptibility testing.

### Ethics Statement

Informed consent was not obtained as included patients were not subjected to extra procedures or questions. No personal information was stored in the study database. As the intervention of this study was purely laboratory based without involvement of patients formal IRB review was not necessary. Samples were collected as part of standard care and samples employed in the analyses were de-identified before access.

**Figure 1:**
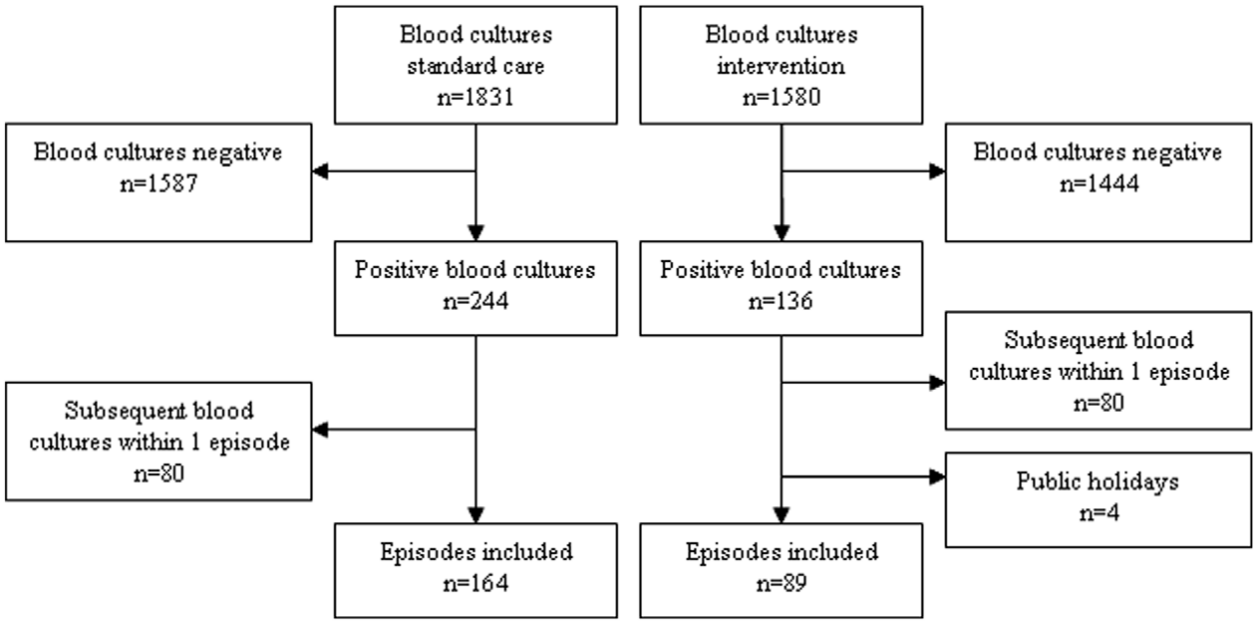
Flowchart of bacteremia episodes included during the intervention and standard care period.

### Preparation of samples

Five ml of a positive blood culture broth was centrifuged at 170 g for 5 minutes. Subsequently, 3 ml of the supernatant was centrifuged at 1500 g for 5 minutes. The pellet was resuspended in 5 ml of distilled water and centrifuged again at 1500 g for 5 minutes. 3 µl of the pellet was resuspended in 25 µl of 70% formic acid and mixed with 25 µl of acetonitrile. After final centrifugation at 18600 g for 2 minutes, 1 µl of the supernatant was spotted in duplicate onto a MALDI-TOF MS MSP 96 ground steel target plate (#224990, Bruker Daltonics, Bremen, Germany). The spots were overlaid with 1 µl of matrix solution (α-cyano-4-hydroxycinnamic acid, #255344, Bruker Daltonics) and air dried.

### Mass spectrometry

Analyses were performed on a microflex LT mass spectrometer (Bruker Daltonics). To identify microorganisms, raw spectra obtained for each isolate were imported into BioTyper software, version 2.0 (Bruker Daltonics), and analysed using default parameter settings. Based on the results of previous studies that showed high percentages of correct identification in isolates with score values above 1.7 [6], [7], isolates with spectral score≥1.7 were considered correctly identified.

### Data collection and statistical analysis

Times of blood culture positivity were recorded by the Bactec 9240 system and identification times were extracted from the laboratory information system. Antibiotic therapy data (including all changes made) were collected from the hospital and intensive care units' information systems. Antimicrobial therapy was considered inappropriate when isolated pathogens were resistant to prescribed antimicrobial agents or when antibiotic therapy was not according to the hospital guidelines. Species identification based on the Phoenix system (Becton-Dickinson) was considered reference standard.

All episodes were analyzed according to intention-to-treat analysis. Primary outcome measure was the proportion of episodes on adequate antimicrobial treatment within 24 hours. Secondary outcome parameters included identification time, time until the first switch in antibiotic treatment and number of switches in therapy.

Results for continuous variables are expressed as means with standard deviation (SD) or as median with interquartile range (IQR) when not normally distributed, and for categorical variables as percentages with the absolute number between parentheses. Summary data were calculated using a Student's t test (normal distribution) or Mann-Whitney U test (skewed distribution) for continuous variables and by chi-square analysis for categorical variables. Statistical analyses were performed with SPSS version 15.0 (Windows, Chicago, USA).

**Table 1:** Number of correct and incorrect direct MALDI-TOF MS identification in 77 monomicrobial episodes.

	Gram positive cocci	Gram positive bacilli	Gram negative bacilli	Total
Correct identification score≥2.0	6 (11.8%)	-	14 (60.9%)	20 (26.0%)
Correct identification 1.7≤score<2.0	21 (41.2%)	2 (66.7%)	6 (26.1%)	29 (37.7%)
No reliable identification	21 (41.2%)	1 (33.3%)	2 (8.7%)	24 (31.2%)
No MALDI-TOF MS	3 (5.9%)		1 (4.3%)	4 (5.2%)
Total	51 (100%)	3 (100%)	23 (100%)	77 (100%)

## Results

There were 253 episodes (218 patients) of bacteremia during the 4-month study period; 89 during the direct MALDI-TOF MS period and 164 during the standard care period. This difference in included episodes between both periods results from differences in the total number of blood cultures submitted to the laboratory (1580 during the intervention period vs 1831 during the control period) and the number of blood cultures that became positive (136 during the intervention period vs 244 during the control period). In addition, no direct MALDI-TOF MS was performed on public holidays (figure 1).

**Table 2:** Number of correct and incorrect direct MALDI-TOF MS identification in 12 polymicrobial episodes.

	Mixed infections, 2 species	Mixed infections, 3 species
Correct identification all microorganisms	1 (10%)	-
Correct identification 1 of the species with score≥2.0	3 (30%)	-
Correct identification 1 of the species with 1.7≤score<2.0	2 (20%)	1 (50%)
No reliable identification of any of the species	4 (40%)	1 (50%)
Total	10 (100%)	2 (100%)

Patients included in both periods were comparable with respect to age (53.0 vs 55.0 for direct MALDI-TOF MS versus standard care respectively, p0.37), proportion of ICU patients (20.2% vs 21.3%, p0.84), proportion of paediatric patients (22.5% vs 19.5%, p0.77) and proportion of contaminated blood cultures (13.5% vs 11.0%, p0.56). Most blood cultures grew Gram-positive cocci (59.7%) or Gram-negative bacilli (25.3%) (table S1). Differences in isolated pathogens between the intervention and standard care were not significant (p0.74).

Direct MALDI-TOF MS analysis yielded correct identification in 56.2% of episodes (n = 50) with spectral score≥1.7. No reliable identification was obtained in 39.3% (n = 35) of episodes (no MALDI-TOF identification (n = 25), identification with score value<1.7 (n = 4) or identification of one species while the blood culture yielded 2 or 3 species (n = 6)). In the remaining 4.5% of episodes, no direct MALDI-TOF MS was performed (n = 4).

Of monomicrobial episodes, 63.6% was correctly identified and of episodes caused by Gram-negative bacilli, 87.0% of all identifications were correct (table 1 and table S2). For most polymicrobial episodes, no reliable identification was obtained (table 2).

Direct MALDI-TOF MS analysis resulted in a 28.8-h reduction in the median time to species identification, from 45.2 hours (IQR 35.5–55.9) during standard care to 16.4 hours (IQR 10.3–42.9) in the intervention period (p<0.001) (table 3). In the direct MALDI-TOF MS group, species identification was available within 10 hours in 23.6% of episodes. Without direct MALDI-TOF MS, in 76.2% of episodes identification was only available after more than 35 hours after growth detection.

**Table 3:** Effect of direct MALDI-TOF MS on identification time and antibiotic switching.

		Direct MALDI-TOF MS (n = 89)	Standard care (n = 164)	p-value
Median identification time in hours (IQR)		16.4 (10.3–42.9)	45.2 (35.5–55.9)	<0.001
Episodes with ID time	<10 h	23.6%	0.6%	<0.001
	10–35 h	44.9%	23.2%	0.001
	35–50 h	16.9%	36.6%	0.001
	>50 h	14.6%	39.6%	<0.001
Median time until first switch in antibiotic therapy in hours (IQR)		17.5 (9.8–38.8)	24.0 (9.5–47.0)	0.30
Number of switches	0	55.0%	58.8%	0.59
	1	41.6%	34.8%	0.28
	2	3.4%	6.7%	0.27
1st switch same day BC^a^ positive		40.0%	29.2%	0.20
1st switch 1 day after BC^a^ positive		30.0%	38.5%	0.47
1st switch>1 day after BC^a^ positive		30.0%	32.3%	0.92

a blood culture.

At the time of blood culture positivity, antimicrobial therapy covered the pathogen(s) isolated from the blood culture in 53.0% (n = 134) of patients (MALDI-TOF MS 55.1%, conventional 51.8%) (p 0.20). In 20.9% (n = 53) of patients antimicrobial therapy was considered inappropriate and in 26.1% (n = 66) of patients blood cultures were considered contaminated or no antimicrobial treatment had been initiated. Inappropriate therapy most frequently occurred for Coagulase-negative staphylococci (n = 12), *S. aureus* (n = 10), enterococci (n = 8) and for Enterobacteriaceae considered as potential AmpC producers (n = 5) (table S3).

Twenty-four hours after blood culture positivity, proportions of appropriate treatment were 64.0% and 75.3% in the standard care and intervention period, respectively (p0.01) (table 4). If a reliable MALDI-TOF MS result was obtained the proportion of appropriate treatment after 24 hours rose to 82.0%.

**Table 4:** Effect of direct MALDI-TOF MS on proportion of appropriate treatment.

	Direct MALDI-TOF MS	Standard care
% (n) of episodes with appropriate therapy<24 h after positive BC^a^	75.3% (67)*	64.0% (105)*
% (n) of episodes with inappropriate therapy<24 h after positive BC^a^	4.5% (4)*	14.6% (24)*
% (n) of episodes without antibiotic therapy<24 h after positive BC^a^	20.2% (18) (6.7% (6) other interventions^b^, 13.5% (12) contaminated BC)	21.4% (35) (4.3% (7) other interventions^b^, 11.0% (18) contaminated BC, 6.1% (10) not applicable^c^)

a blood culture, ^b^removal of intravenous catheters, ^c^palliative care or patient died shortly after blood culture was positive.

*p value 0.01.

Median times until the first switch in antibiotic therapy were 17.5 hours (IQR 9.8–38.8) and 24.0 hours (IQR 9.5–47.0) in the intervention and standard care periods, respectively (p0.30) (table 3). In 57.3% of bacteremia episodes antibiotic therapy was not changed (intervention 55.0%, standard care 58.8%). The number of antibiotic changes and the time till change were not statistically significant between both study groups (data not shown).

## Discussion

In this trial of 253 episodes of bloodstream infections, MALDI-TOF MS directly performed on positive blood culture broths reduced the time until definitive identification of bacterial species by 28.8 hours and increased the proportion of patients on appropriate antimicrobial therapy within 24 hours by 11.3%.

In previous studies, the clinical impact of rapid microbiology results has been investigated by means of different interventions, including the immediate incubation of blood cultures outside the laboratory operation time [10], reducing turnaround time by the use of the Microscan system (Baxter-Microscan, Sacramento, USA) [11], more rapid reporting of culture results [12] and direct analysis of positive blood cultures with the Vitek System (bioMerieux, Marcy-l'Etoile, France) in combination with more rapid reporting of culture results [13]. As a consequence of these interventions, shorter time until the first switch in antibiotic treatment [10], shorter hospital stay [12], lower mortality [11], lower antibiotic use [13] and lower costs [11], [12] were reported.

Most of these studies examined the clinical effect of more rapid identification in combination with rapid susceptibility testing [10]–[12], and only one of these studies was restricted to blood culture isolates [10]. Moreover, the exact time of antibiotic switching was not recorded in all studies and different definitions of switching were used [12], [13]. In this study only the identification time was shortened while the duration of susceptibility testing was left unchanged. Therefore, any change in the proportion of patients on adequate treatment can be attributed to the use of direct MALDI-TOF MS analysis.

Contribution of microbiology results to antimicrobial management depends on local antimicrobial policies and bacterial ecology. This study was performed in a setting where antibiotic resistance is infrequent, which allows species identification to be a more powerful tool than in settings with more complex resistance patterns. Inadequate empirical therapy occurs infrequently in our population because of low resistance levels. For instance, there were no bacteremia episodes with MRSA and vancomycin resistant enterococci and empirical therapy was adequate in the majority of patients already receiving antimicrobial treatment.

In this study antimicrobial therapy was judged inappropriate when isolated pathogens were resistant to prescribed agents or when antibiotic therapy was not according to the hospital guidelines. Consequently, a few antimicrobial regimens judged inappropriate may, although suboptimal, be effective against the causative pathogens. In this study, direct MALDI-TOF MS yielded correct identification in a lower proportion of episodes compared to the results of previous studies. This might be explained by a relatively large proportion of Gram positive and polymicrobial infections in this study, while in other studies polymicrobial samples have been excluded[6]. It is known that Gram positive and polymicrobial infections are often not accurately identified by direct MALDI-TOF MS [14]. However, adaptations in sample processing may increase rates of correctly identified microorganisms [7], [15]–[17] and thereby increase clinical impact of direct MALDI-TOF MS even further.

Limitations of this study include the fact that the study size was too small to evaluate differences in morbidity or mortality between the intervention group and the standard care group. In addition, MALDI-TOF MS only provides identification without information on antimicrobial susceptibility. This information can guide antibiotic management better in a setting with low levels of antibiotic resistance compared to areas with higher levels of resistance. This may decrease generalizability of our data to countries with higher levels of antibiotic resistance.

In conclusion, the use of direct MALDI-TOF MS on positive blood cultures resulted in a faster identification of microorganisms causing bloodstream infection. Routine implementation of this technique increased the proportion of episodes on adequate antimicrobial treatment within 24 hours. The cost effectiveness of this procedure remains to be determined.

## Supporting Information

Table S1 **Number of microorganisms identified from episodes of positive blood cultures throughout the study period.**
(DOC)

Table S2 **Conventional and direct MALDI-TOF MS identification of isolated pathogens from 77 monobacterial samples.**
(DOC)

Table S3 **Antimicrobial therapy considered inappropriate.**
(DOC)
