# Supplementary material for: Direct Matrix-Assisted Laser Desorption Ionization Time-of-Flight Mass Spectrometry Improves Appropriateness of Antibiotic Treatment of Bacteremia
Source: PLoS One. 2012 Mar 16;7(3):e32589. doi: 10.1371/journal.pone.0032589 (PMC3306318; doi:10.1371/journal.pone.0032589)
Supplement: Table S1 — Number of microorganisms identified from episodes of positive blood cultures throughout the study period. (DOC) [file pone.0032589.s001.doc]

Supporting table 1.

| MICROORGANISM(S) | | DEC | FEB | MRCH | APR | TOTAL |
| --- | --- | --- | --- | --- | --- | --- |
| **Gram positive cocci** | | **58** | **34** | **42** | **17** | **151** |
|  | Coagulase negative staphylococcus | 30 | 19 | 18 | 12 | 79 |
|  | Staphylococcus aureus, methicillin-sensitive | 6 | 6 | 11 | 2 | 25 |
|  | Micrococcus species | 1 |  | 1 |  | 2 |
|  | Streptococcus haemolyticus Group A |  | 1 | 2 |  | 3 |
|  | Streptococcus haemolyticus Group B |  | 1 |  |  | 1 |
|  | Streptococcus haemolyticus Group G | 3 |  |  |  | 3 |
|  | Streptococcus intermedius |  |  | 1 |  | 1 |
|  | Streptococcus species | 1 |  |  |  | 1 |
|  | Streptococcus pneumoniae | 3 | 4 | 2 | 1 | 10 |
|  | Streptococcus salivarius | 1 |  |  |  | 1 |
|  | Viridans streptococcus | 6 | 1 | 2 |  | 9 |
|  | Streptococcus mitis |  |  |  | 1 | 1 |
|  | Enterococcus species | 1 | 1 | 3 |  | 5 |
|  | Amoxicillin resistent Enterococcus faecium | 5 | 1 | 1 | 1 | 8 |
|  | Enterococcus cecorum | 1 |  |  |  | 1 |
|  | Abiotrophia defectiva |  |  | 1 |  | 1 |
|  |  |  |  |  |  |  |
| **Gram positive bacilli** | | **3** | **2** | **1** | **1** | **7** |
|  | Corynebacterium species | 1 |  |  |  | 1 |
|  | Bacillus cereus |  | 1 |  |  | 1 |
|  | Bacillus species | 1 |  |  |  | 1 |
|  | Listeria monocytogenes |  |  |  | 1 | 1 |
|  | Propionibacterium species | 1 | 1 |  |  | 2 |
|  | Propionibacterium acnes |  |  | 1 |  | 1 |
|  |  |  |  |  |  |  |
| Gram negative cocci | |  |  | 1 |  | 1 |
|  | Moraxella osloensis |  |  | 1 |  | 1 |
|  |  |  |  |  |  |  |
| **Gram negative bacilli** | | **21** | **10** | **21** | **12** | **64** |
|  | Escherichia coli | 7 | 2 | 11 | 8 | 28 |
|  | Klebsiella pneumoniae | 3 | 2 | 2 |  | 7 |
|  | Klebsiella oxytoca | 1 |  |  | 1 | 2 |
|  | Proteus mirabilis | 1 |  | 2 |  | 3 |
|  | Serratia marcescens | 1 | 1 |  |  | 2 |
|  | Enterobacter cloacae |  | 2 | 1 | 1 | 4 |
|  | Enterobacter hormaechei | 1 |  |  |  | 1 |
|  | Citrobacter freundii | 1 |  |  |  | 1 |
|  | Morganella morganii |  |  | 1 |  | 1 |
|  | Salmonella enteritidis | 1 |  |  |  | 1 |
|  | Pseudomonas aeruginosa | 4 |  | 1 | 1 | 6 |
|  | Bacteroides species | 1 | 3 | 3 | 1 | 8 |
|  |  |  |  |  |  |  |
| **Yeast** | | **2** |  | **1** |  | **3** |
|  | Candida albicans |  |  | 1 |  | 1 |
|  | Candida krusei | 1 |  |  |  | 1 |
|  | Candida parapsilosis | 1 |  |  |  | 1 |
|  |  |  |  |  |  |  |
| **Mixed infections** | | **5** | **7** | **9** | **6** | **27** |
|  | Amoxicilline resistente Enterococcus faecium + Coagulase negative staphylococcus | 1 |  |  |  | 1 |
|  | Corynebacterium species + Coagulase negative staphylococcus | 1 |  |  |  | 1 |
|  | Streptococcus pneumoniae + Coagulase negative staphylococcus | 1 |  |  |  | 1 |
|  | Streptococcus species + Nonfermentative gram negative bacillus | 1 |  |  |  | 1 |
|  | Citrobacter youngae + Staphylococcus aureus, methicillin-sensitive | 1 |  |  |  | 1 |
|  | Coagulase negative staphylococcus + Coagulase negative staphylococcus |  | 1 | 2 | 1 | 4 |
|  | Amoxicillin resistent Enterococcus faecium + Coagulase negative staphylococcus |  | 1 |  |  | 1 |
|  | Escherichia coli + Enterococcus species |  | 1 | 1 |  | 2 |
|  | Streptococcus pneumoniae + Coagulase negative staphylococcus |  | 1 |  |  | 1 |
|  | Streptococcus haemolyticus Group C + Staphylococcus aureus, methicillin-sensitive |  | 1 |  |  | 1 |
|  | Scedosporium species + Coagulase negative staphylococcus |  |  | 1 |  | 1 |
|  | Klebsiella pneumoniae + Enterococcus species |  |  | 1 |  | 1 |
|  | Staphylococcus aureus, methicillin-sensitive + Coagulase negative staphylococcus |  |  | 2 |  | 2 |
|  | Stenotrophomonas maltophilia + Streptococcus species |  |  | 1 |  | 1 |
|  | Stenotrophomonas maltophilia + Viridans streptococcus |  |  | 1 |  | 1 |
|  | Enterobacter cloacae + Clostridium perfringens |  |  |  | 1 | 1 |
|  | Escherichia coli + Escherichia coli |  |  |  | 1 | 1 |
|  | Enterococcus species + Coagulase negative staphylococcus |  |  |  | 2 | 2 |
|  | Enterococcus species + Streptococcus species |  |  |  | 1 | 1 |
|  | Candida albicans + Streptococcus anginosus + Escherichia coli |  | 1 |  |  | 1 |
|  | Coagulase negative staphylococcus + Coagulase negative staphylococcus + Pseudomonas orizyhabitans |  | 1 |  |  | 1 |
|  |  | **89** | **53** | **75** | **36** | **253** |
