# Supplementary material for: Direct Matrix-Assisted Laser Desorption Ionization Time-of-Flight Mass Spectrometry Improves Appropriateness of Antibiotic Treatment of Bacteremia
Source: PLoS One. 2012 Mar 16;7(3):e32589. doi: 10.1371/journal.pone.0032589 (PMC3306318; doi:10.1371/journal.pone.0032589)
Supplement: Table S2 — Conventional and direct MALDI-TOF MS identification of isolated pathogens from 77 monobacterial samples. (DOC) [file pone.0032589.s002.doc]

Supporting table 2.

| **Conventional identification (n)** | **MALDI-TOF identification (n)** | **Correct identification at species level % (n)** | **Correct identification at genus level % (n)** |
| --- | --- | --- | --- |
| Enterococcus faecium (2) | Enterococcus faecium (2) | 100% (2) | - |
| Bacillus cereus (1) | Bacillus species | - | 100% (1) |
| Bacteroides species (4) | Bacteroides fragilis (2)  No reliable identification (1)  No MALDI-TOF performed (1) | - | 50% (2)^1^ |
| Coagulase negative staphylococcus (31) | Staphylococcus haemolyticus (3)  Staphylococcus capitis (2)  Staphylococcus hominis (3)  Staphylococcus epidermidis (6)  Staphylococcus condimenti (1)  No reliable identification (14)  No MALDI-TOF performed (2) | - | 48.4% (15)^1^ |
| Enterobacter cloacae (3) | Enterobacter cloacae (2)  Enterobacter species (1) | 66.7% (2) | 33.3% (1) |
| Enterococcus species (1) | Enterococcus faecalis (1) | - | 100% (1)^1^ |
| Escherichia coli (11) | Escherichia coli (11) | 100% (11) | - |
| Klebsiella oxytoca (1) | Klebsiella oxytoca (1) | 100% (1) | - |
| Klebsiella pneumoniae (2) | Klebsiella pneumoniae (2) | 100% (2) | - |
| Listeria monocytogenes (1) | Listeria monocytogenes (1) | 100% (1) | - |
| Propionibacterium species (1) | No reliable identification (1) | - | - |
| Pseudomonas aeruginosa (1) | No reliable identification (1) | - | - |
| Serratia marcescens (1) | Serratia marcescens (1) | 100% (1) | - |
| Staphylococcus aureus (8) | Staphylococcus aureus (4)  No reliable identification (4) | 50% (4) | - |
| Streptococcus haemolyticus Group A (1) | No MALDI-TOF performed (1) | - | - |
| Streptococcus haemolyticus Group B (1) | Streptococcus agalactiae (1) | 100% (1) | - |
| Streptococcus mitis (1) | No reliable identification (1) | - | - |
| Streptococcus pneumoniae (5) | Streptococcus pneumoniae (3)  No reliable identification (2) | 60% (3) | - |
| Viridans streptococcus (1) | Streptococcus bovis (1) | - | 100% (1)^1^ |

^1^conventional identification does not provide identification at the species level
