# Supplementary material for: Direct Matrix-Assisted Laser Desorption Ionization Time-of-Flight Mass Spectrometry Improves Appropriateness of Antibiotic Treatment of Bacteremia
Source: PLoS One. 2012 Mar 16;7(3):e32589. doi: 10.1371/journal.pone.0032589 (PMC3306318; doi:10.1371/journal.pone.0032589)
Supplement: Table S3 — Antimicrobial therapy considered inappropriate. (DOC) [file pone.0032589.s003.doc]

Supporting table 3.

| Micro-organism(s) | Antimicrobial treatment at time of blood culture positivity |
| --- | --- |
| Coagulase negative staphylococcus (Oxacillin R) | Flucloxacillin (n=1)  Augmentin (n=2)  Imipenem (n=2)  Meropenem (n=2)  Ceftriaxone (n=2)  Cefazolin (n=1) |
| Coagulase negative staphylococcus (Oxacillin S) | Imipenem (n=1)  Meropenem (n=1) |
| Staphylococcus aureus | Ciprofloxacin (n=1)  Ceftriaxone (n=7)  Augmentin (n=2)  Cefazolin (n=1)  Ceftazidim + Vancomycin (n=1) |
| Enterococcus faecium (Amoxicillin R) | Piperacillin-tazobactam (n=1)  Augmentin (n=1)  Imipenem (n=2)  Ceftriaxone (n=1)  Ceftriaxone + Metronidazole (n=1) |
| Enterococcus cecorum | Meropenem (n=1) |
| Enterococcus species | Ceftriaxone (n=1) |
| Streptococcus salivarius | Ciprofloxacin (n=1) |
| Viridans streptococcus | Flucloxacillin (n=1) |
| Streptococcus pneumoniae | Ciprofloxacin (n=1) |
| Escherichia coli (Amoxicillin-clavulanate R) | Amoxicillin-clavulanate (n=1) |
| Citrobacter freundii | Ceftriaxone (n=1) |
| Serratia marcescens | Ceftriaxone (n=1) |
| Morganella morganii | Ceftriaxone (n=1) |
| Enterobacter cloacae | Ceftriaxone (n=1)  Flucloxacillin (n=1) |
| Enterobacter hormachei | Ceftriaxone (n=1) |
| Stenotrophomonas maltophilia | Ceftriaxone (n=1) |
| Bacteroides species | Ceftriaxone + Ciprofloxacin (n=1) |
| Coagulase negative staphylococcus (Oxacillin R, Clindamycin R) + Enterococcus faecium (Amoxicillin R) | Ceftriaxone + Clindamycin (n=2) |
| Enterococcus faecium (Amoxicillin R) + Coagulase negative staphylococcus (Oxacillin R) | Meropenem (n=1) |
| Enterococcus species + Coagulase negative staphylococcus (Oxacillin R) | Ceftriaxone (n=1) |
| Staphylococcus aureus + Streptococcus haemolyticus Group C | Cefazolin + Gentamicin (n=1) |
| Escherichia coli (Ceftriaxone R) + Enterococcus species | Ceftriaxone (n=1) |
| Candida krusei (high MIC amfotericin B) | Amfotericin B (n=1) |
| Candida albicans + Escherichia coli + Streptococcus anginosus | Ceftriaxone (n=1) |
